# Adenosine A_2A_ receptors in the rostral ventrolateral medulla participate in blood pressure decrease with electroacupuncture in hypertensive rats

**DOI:** 10.3389/fcvm.2023.1275952

**Published:** 2023-10-19

**Authors:** Zhi-Ling Guo, Stephanie C. Tjen-A-Looi, Anh Thingoc Nguyen, Liang-Wu Fu, Hou-Fen Su, Yiwei D. Gong, Shaista Malik

**Affiliations:** Susan-Samueli Integrative Health Institute and Department of Medicine, College of Health Sciences, University of California, Irvine, CA, United States

**Keywords:** peripheral nerve stimulation, acupuncture, brain stem, adenosine, sympathoexcitation

## Abstract

Acupuncture is increasingly used to manage high blood pressure (BP) as a complementary therapy. However, the mechanisms underlying its hypotensive effects remain unclear. Our previous studies have shown that electroacupuncture (EA) at the ST36-37 acupoints, overlying the deep peroneal nerve, attenuates pressor responses through adenosine A_2A_ receptors (A_2A_R) in the rostral ventrolateral medulla (rVLM). However, it is uncertain whether rVLM A_2A_R contributes to EA's BP-lowering effect in sustained hypertension. We hypothesized that a course of EA treatment lowers BP, in part, through the activation of adenosine A_2A_R in the rVLM in hypertensive rats. To mimic essential hypertension in the clinic, we performed EA in conscious Dahl salt-sensitive hypertensive rats (DSHRs). EA (0.1–0.4 mA, 2 Hz) was applied at ST36-37 for 30 min twice weekly for four weeks, while sham-EA was conducted in a similar manner but without electrical input. In hypertensive rats, BP was reduced by EA (*n* = 14) but neither by sham-EA (*n* = 14) nor in the absence of needling (*n* = 8). Following four weeks of eight treatments and then under anesthesia, EA's modulatory effect on elevated BP was reversed by unilateral rVLM microinjection of SCH 58261 (1 mM in 50 nl; an A_2A_R antagonist; *n* = 7; *P* < 0.05) but not the vehicle (*n* = 5) in EA-treated DSHRs. Activation of rVLM A_2A_R in DSHRs treated with sham-EA by an A_2A_R agonist, CGS-21680 (0.4 mM in 50 nl; *n* = 8), decreased BP. Unilateral administration of SCH 58261 or CGS-21680 into the rVLM did not alter basal BP in Dahl salt-sensitive rats fed a regular diet with normal BP. The A_2A_R level in the rVLM after EA was increased compared to the sham-EA and untreated DSHRs (*n* = 5 in each group; all *P *< 0.05). These data suggest that a 4-week twice weekly EA treatment reduced BP in salt-sensitive hypertensive rats likely through adenosine-mediated A_2A_R in the rVLM.

## Introduction

Hypertension and its consequences, such as stroke and heart attacks, are enormous public health problems, particularly in the aged population (1). Approximately one in three adults have high blood pressure (BP) worldwide. However, about half of the patients with hypertension struggle with achieving normal BP levels due to many factors, including concerns about side effects from medication (2). Non-pharmacologic procedures offer a complementary therapy to manage hypertension (3, 4). As such, an urgent need for non-pharmacological effective therapies is warranted. Acupuncture, including manual and electroacupuncture, have been used empirically to treat hypertension for many years in Eastern Asia (5). In recent years, studies have reported that acupuncture offers the potential to manage cardiovascular disorders, including hypertension, in the Western world (3, 4). This therapy has been used as a complemental and integrative regimen (5). However, the specific mechanisms underlying the antihypertensive effect of acupuncture need to be further elucidated for its broad clinical application.

We have studied the neural mechanisms underlying acupuncture's effects on elevated BP. In this regard, our previous studies have shown that through rostral ventrolateral medulla (rVLM) electroacupuncture (EA) at the ST36-37 acupoints lowers both sympathoexcitatory BP reflex response and sustained elevation of BP in cold-induced hypertensive rats associated with a significant increase in sympathetic nerve activity (4, 6–8). The EA's BP-lowering effect following completion of the therapy involves increased expression of pre-proenkephalin mRNA and activation of opioid receptors in the rVLM (4, 9). This region in the brainstem plays an essential role in regulating sympathetic outflow leading to BP control (10). As such, the rVLM is critical in the BP-modulatory effect of EA, including the lowering of both sympathoexcitatory cardiovascular reflex responses and chronic over-excitation of sympathetic activity in hypertension.

The purinergic (P) system has been associated with the regulation of the cardiovascular system. Adenosine produced during the metabolism of adenosine-5-triphosphate (ATP) is released by neuronal activation (11, 12). While ATP activates neurons by stimulating P2 receptors, adenosine primarily modulates neuronal function through P1 (subclassified into A_1_, A_2_, and A_3_) receptors in the nervous system, including central control of autonomic function (13–15). In this regard, we recently have demonstrated that in addition to opioids, adenosine through the A_2A_ receptors (A_2A_R) in the rVLM participates in EA modulation of cardiovascular excitatory BP reflex responses in the normotensive rat (13). Other investigators have reported that the administration of adenosine, through an A_2A_R mechanism in the nucleus tractus solitarius (NTS) and area postrema, lowers the BP of hypertensive rats (15, 16) suggesting a potential BP-lowering effect induced by enhanced activation of the A_2A_R in the rVLM in hypertensive subjects. Consequently, we investigated the non-pharmacological activation of rVLM A_2A_R by EA at ST36-37 in hypertensive rats. We aimed to determine if rVLM A_2A_R contributes to the decrease in BP following the completion of the EA treatment in hypertensive rats.

To mimic essential hypertension commonly related to genetic and environmental factors, we selected Dahl salt-sensitive hypertensive rats (DSHRs). This animal model is used to study hypertension addressing neurogenic cardiovascular features observed in essential hypertension (17, 18), while elevated sympathetic activity, associated with hypertension (17, 18), is decreased by EA (5, 19, 20).

## Materials and methods

The study conformed to the American Physiological Society's Guiding Principles for Research Involving Animals and Human Beings. Experimental protocols were approved by the Institutional Animal Care and Use Committee of the University of California at Irvine. Male Dahl salt-sensitive (DS) rats (280–300 g) were purchased from Charles River Laboratories International, Inc.). Previous studies have demonstrated that DS rats fed a regular diet do not develop hypertension (21–23). Thirty-six DS rats were fed 4% high salt (4% NaCl) to induce hypertension. To evaluate the developing salt-sensitive hypertension, 13 other DS rats on a regular diet served as normotensive controls. All DS rats were given tap water *ad libitum* and housed in a 12–12 h light-dark cycle.

**Figure 1:**
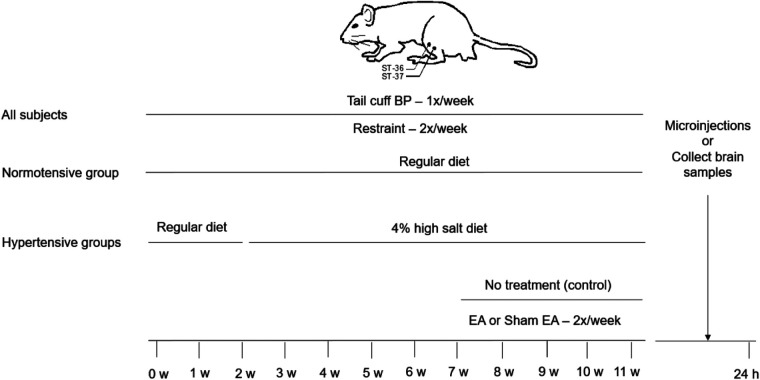
Timeline of experiments for four groups of rats. The Dahl salt-sensitive (DS) rats were randomly divided into normotensive control, EA, sham-EA, and untreated hypertension groups. All DS rats were restrained for 30 min twice weekly throughout the experiment. Normotensive control rats were fed a regular diet over the experimental period. In hypertensive groups, two weeks after restrained training, DS rats were given the 4% high salt diet in the three groups for nine weeks. After feeding with a 4% high salt diet for five weeks, the DS rats were treated with EA, sham-EA at the ST36-37 acupoints, or restraint only without the needle insertion for 30 min, twice weekly for another four weeks in one of these three groups, respectively. Blood pressure (BP) in all group rats was evaluated weekly using a tail-cuff device. Twenty-four hours after terminating the treatment, the rVLM microinjections were performed in rats treated with EA, sham-EA, or normal controls to examine a role of A_2A_ receptors (A_2A_R) in the rostral ventrolateral medulla (rVLM)”. Besides, rats from each hypertensive group were decapitated to examine the expression of A_2A_R in the rVLM.

After one week of conditioning to familiarize animals with the handling, all DS rats were gently handled and restrained in slings for 30 min twice weekly, in the morning (9:00–12:00 PM) on Tuesday and Thursday, throughout the 11-week experimental period (Figure 1). Each rat was wrapped with a cloth around the body, excluding the four limbs. These procedures prevent stress associated with experimental interventions in EA, Sham-EA, and hypertensive and normotensive control rats (4, 23). Also, this method allowed the application of EA at the ST36-37 acupoints safely and effectively in the immobilized conscious rats. Acupuncture needles (40-gauge stainless steel; 0.16 mm diameter) were inserted into ST36-37 briefly (∼10 s) in DS rats fed with 4% high salt to allow them to become accustomed to handling and needling prior to the four weeks of EA therapy. The ST36-37 acupoints in the rat, analogous to those in humans, are located 5 mm lateral to the midline of the tibia's anterior tubercle and correspondingly 10 mm and 15 mm below the center of the knee joint of the hind limb (overlying deep peroneal nerve) (4, 24, 25).

BP in conscious rats was measured non-invasively with a volume pressure recording sensor and an occlusion tail-cuff (CODA System, Kent Scientific) once a week, every Friday morning (9:00 AM–12:00 PM), as we and others have described previously (4, 22, 23). In brief, each animal was placed in a restrainer, and the cuff placed on the tail was inflated and released several times to condition the rat for the procedure. After stabilization, BP was measured five times to acquire an average value. BPs in all rats were recorded and evaluated weekly throughout the experiment.

### Experimental protocols

Normotensive control group: DS rats were fed a regular diet for 11 weeks with 30 min restraint twice weekly. No acupuncture was applied to this group since EA does not alter BP in normotensive conditions (26–28).

The rats fed with a high salt diet demonstrated an elevated BP after five weeks. Dahl salt-sensitive hypertensive rats (DSHRs) were then divided randomly into the following three groups (Figure 1).

(1) EA-treated group: To stimulate acupoints ST36-37, conscious rats were wrapped in slings accommodating for accessibility to the hindlimbs (4, 28). Pairs of sterile acupuncture needles were inserted perpendicularly through the skin bilaterally into ST36-37 at a depth of 5 mm (4, 29). The needles were connected to a photoelectric stimulus isolation unit and the stimulator (model no. S88, Grass, West Warwick, RI, United States). Each set of electrodes at ST36-37 on each limb was stimulated separately with a positive and negative pole to prevent current flowing from one location to the contralateral hindlimb. To confirm the proper placement of acupuncture needles at ST36-37, we applied a current (0.6–1.0 mA) to these acupoints to observe slight repetitive paw twitches. The twitches indicated the proper stimulation of motor fibers in the mixed nerve bundle comprising the deep peroneal nerve. Subsequently, we adjusted the current to a low intensity (i.e., 0.3–0.5 mA) with noticing very slight flexing of the digits of the paw to administer EA treatment (2 Hz, 0.5 ms duration). Sensory but not motor nerve activation contributes to EA-inhibitory cardiovascular responses (5, 30). The specific parameters of EA stimulation at ST36-37 were similar to our previous study, shown to stimulate sensory afferents (both finely myelinated and unmyelinated fibers), and reduce reflex-induced elevation in BP and high BP in the rat model of sustained hypertension (4, 6, 31, 32). EA was applied for 30 min, twice weekly for four weeks (4).
(2) Sham-EA group: DSHRs received the same treatment as those in the EA group, except needles were inserted at ST36-37 but were not electrically stimulated. In this study, it was an appropriate control for the electroacupuncture study (4, 32).
(3) Hypertensive control group: DSHRs were restrained for 30 min twice a week for 11 weeks but were not subjected to the insertion of acupuncture needles for the treatment.

### Microinjection into the rVLM

To examine the role of rVLM A_2A_R in EA modulation of elevated BP, we performed the microinjection of an agonist or antagonist of A_2A_R into the rVLM of DSHRs and normotensive DS rats under anesthesia 24 h after the end of the 4-week treatment with EA, sham-EA, or normotensive controls, as we have described previously (4, 13). In brief, the rat was anesthetized with ketamine (100 mg/kg, i.m.) and *α*-chloralose (50–60 mg/kg, i.v.). Additional doses of *α*-chloralose (25–30 mg/kg, i.v.) were administered to maintain an adequate level of anesthesia by observing the absence of conjunctival reflex response. A femoral vein and artery were cannulated for administrating fluids and monitoring BP, respectively. Heart rate (HR) was derived from the pulsatile BP signal. The trachea was intubated to provide artificial ventilation using a respirator (model 661, Harvard Apparatus). Arterial blood gases were measured with a blood gas analyzer (ABL5, Radiometer America) and maintained within the normal physiological range (PO2 >100 mmHg, PCO2 30–40 mmHg, and arterial pH 7.35–7.4). The body temperature was maintained between 36 and 38°C with a heating pad.

Animals were placed in a stereotaxic head frame. A partial craniotomy was performed to expose the dorsal medulla. One CMA microdialysis probe (14 mm long with 0.24 mm of tip diameter) used for microinjection was modified by removing the microdialysis membrane (13). It was placed unilaterally into the rVLM with coordinates 1.8–2.3 mm lateral from the midline, 1.9–2.2 mm rostral to the obex, and advanced ventrally 3.0–3.3 mm (13, 33, 34). Proper positioning of probes in the rVLM was confirmed by noting a 5–10 mmHg elevation in BP following the probe insertion as well as microinjection of glutamate (2 nmol in 50 nl) (13). The probe was connected to a microsyringe fastened to a microdialysis pump (CMA/102, North Chelmsford, MA, United States) through fluorinated ethylene propylene tubing (0.12-mm inner diameter) and tubing adaptors. The injection of the lowest possible volume of 50 nl was carried out at a rate of 0.6 *μ*l/min over a 5-s period. The injection was administered unilaterally since this approach has been proven successful in maintaining optimal physiological conditions and observing significant responses following the delivery of drugs (6, 13).

#### Drugs

Adenosine A_2A_R agonist, CGS-21680 (0.4 mM) (35); adenosine A_2A_R antagonists, SCH 58261 (1 mM) (36, 37), and the vehicle for these drugs, dimethylsulfoxide (DMSO). All chemicals were purchased from Sigma Aldrich (St. Louis, MO, United States). Affinities, specificities, and dosages for each drug are documented in the cited references. The solution of each drug or vehicle contained 0.5% pontamine sky blue for histological examination of the injection site after the experiment. Microinjection of the vehicle into the rVLM and the drugs into surrounding regions of the rVLM provided chemical and anatomical controls.

#### Protocols of microinjections

(1) SCH 58261 (an A_2A_R antagonist) was microinjected into the rVLM of EA-treated DSHRs (*n* = 9). The vehicle (DMSO) was administered into other sites of the rVLM in random order in five of these nine animals. (2) The rVLM of DSHRs treated with sham-EA (*n* = 9) was administered with CGS-21680 (an A_2A_R agonist). The vehicle (DMSO) was randomly microinjected into the different rVLM sites in five of the nine rats. (3) In separate rats in normotensive groups, SCH 58261 and its vehicle (*n* = 4) or CGS-21680 and its vehicle (*n* = 4) were microinjected into different sites of the rVLM in random order.

#### Histology

Pontamine sky blue (0.5%) was microinjected, along with the chemicals tested in each experiment to mark the injection site. After fixing with 4% paraformaldehyde, coronal sections of the medulla oblongata (60 μm), including the rVLM, were cut with a cryostat. Sites of injection identified using the rat atlas (33) were reconstructed from dye spots (13).

### Western blotting

A_2A_R protein expression in the rVLM was examined from animals in three groups of DSHRs (*n* = 5 in each group) using Western blotting (32). The rVLM tissue was collected 24 hr after four weeks of treatment with EA, sham-EA, and hypertensive controls, as described previously (4, 28). In brief, all animals were anesthetized with a large dose of ketamine/xylazine (100/10 mg/kg, im) and then decapitated. Brain tissue was removed and frozen on dry ice. The rVLM tissue was punched out from 0.5 mm lateral to the lateral edge of the pyramid tract next to the caudal border of the trapezoid body from the ventral surface of the brain stem (4, 28). Bilateral rVLM tissue samples were collected from each rat.

The rVLM tissue was lysed with cell lysis buffer (Cell Signaling Technology, Danvers, MA), which included 137 mM NaCl, 20 mM Tris-HCl (pH 7.5), 10% glycerol, 1% Triton X-100, 0.5% Nonidet P-40, 2 mM EDTA (pH 8.0), 3 μg/ml aprotinin, 3 μg/ml leupeptin, 2 mM phenylmethylsulfonyl fluoride, 20 mM NaF, 10 mM sodium pyrophosphate, and 2 mM Na_3_VO_4_. The protein concentration of each sample (supernatant) was determined using the Bio-Rad Protein Assay (Bio-Rad Laboratories, Hercules, CA). Equal amounts of protein samples were separated by electrophoresis with SDS on 4%–15% polyacrylamide gradient gel (CriterionTM Precast Gel, Bio-Rad Laboratories). The proteins were then transferred to a polyvinylidene difluoride membrane (Millipore, Bedford, MA) and incubated with a blocking buffer (5% nonfat milk in 20 mM Tris-HCl with pH 7.5, 137 mM NaCl, and 0.1% Tween 20) for 1 h at room temperature. The membrane was incubated at 4°C overnight with primary antibodies, including rabbit anti-A_2A_R (1:200; Santa Cruz Biotechnology, Inc., Santa Cruz, CA, United States), or goat anti-*β*-actin (1:200; Santa Cruz Biotechnology) antibodies. The membrane was then washed three times using a solution containing 20 mM Tris-HCl (137 mM NaCl and 0.1% Tween 20; pH 7.5) and incubated with secondary antibodies (1:5,000–1:10,000 dilution) for 1 h at room temperature. The secondary antibodies were rhodamine-conjugated antibodies, including goat anti-rabbit (red, IRDye 680CW, 926-68071) and donkey anti-goat (red, IRDye 800CW, 926-68704; all from LI-COR Biotechnology, Lincoln, NE). After being washed three times, the membrane was detected with Odyssey Imaging System (LI-COR Biotechnology). All comparisons were made with samples run on the same gel and examined on the same film. For the final analysis, the intensity of signals was normalized to the control sample on the same gel. Data were analyzed with ImageJ (NIH). A_2A_R was detected in rVLM, with *β*-actin as the loading control (32).

### Statistical analyses

Data were expressed as means ± SE. Normal data distribution was determined by the Shapiro–Wilk test. Data not normally distributed were analyzed non-parametrically. The difference in BP between the two groups at multiple times was compared using a two-way repeated measure ANOVA followed by the Holm–Sidak method. A one-way repeated measure of ANOVA followed *post hoc* by the Student-Newman Keuls test was used to compare changes in BPs over time as well as before and after drug microinjection in each group. Comparisons of protein expression between two groups were statistically analyzed by the Student's *t*-test. Statistical analyses were performed with Sigma Plot (Jandel Scientific). A significant difference in values was considered when *P *< 0.05.

## Results

### EA’s modulatory effects on elevated BP in hypertensive rats

**Figure 2:**
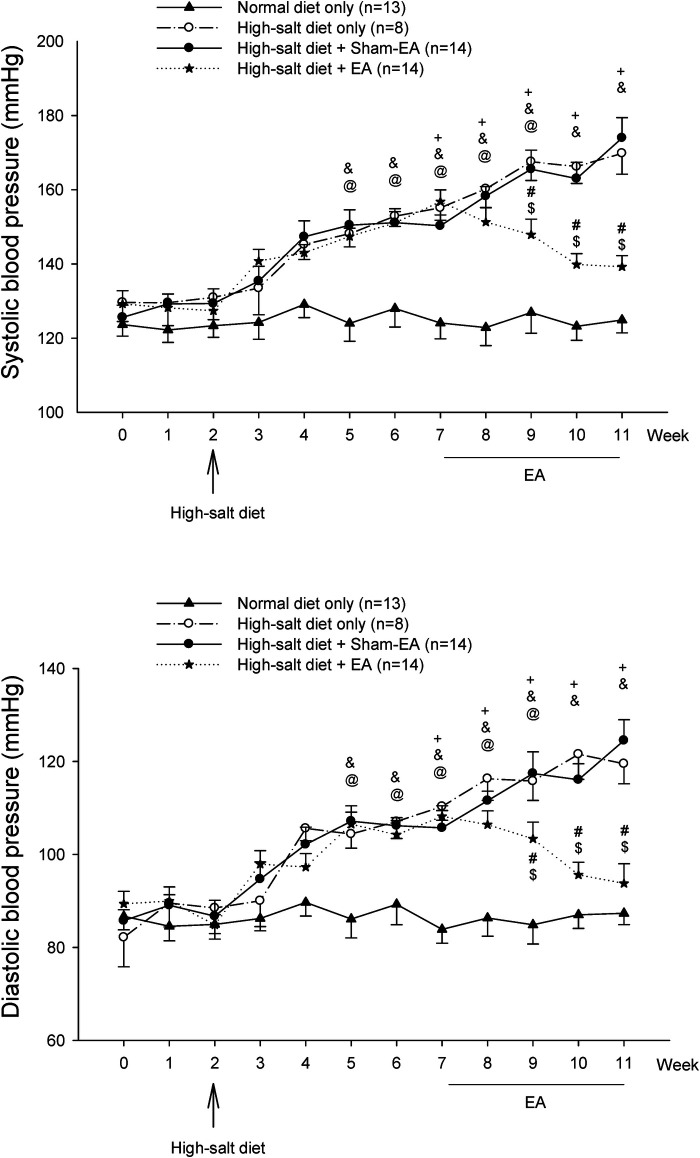
Blood pressure (BP) in Dahl salt-sensitive (DS) rats. Hypertension was developed similarly five weeks after a 4% high salt diet in all three hypertensive groups. Systolic BP (SBP) and diastolic BP (DBP) of DS rats remained elevated for another four weeks in the non-treatment and sham-EA groups. There was no difference in SBP and DBP between these two groups. In contrast, elevated SBP and DBP in the EA-treated group were significantly decreased after two weeks of EA treatment and remained reduced during the remaining course of EA therapy. SBP and DBP of DS rats fed with a regular diet were unaltered throughout the 11 weeks. Values represent means ± SE. The symbols +, &, and @ indicate *P* < 0.05, after vs. before onset of consumption of the high salt diet in hypertension control (o), sham-EA (●), and EA (*), respectively. # and $ correspondingly show *P* < 0.05, EA vs. hypertension control and sham-EA. BPs in three hypertensive groups were higher than in normotensive controls from week 5–week 11 (all *P* < 0.05).

Figure 2 shows BP, including systolic BP (SBP) and diastolic BP (DBP), in four experimental groups of DS rats throughout the 11 weeks. The BPs in the DS rats fed with a regular diet stayed normal and stable over the 11 weeks (122–129/84–87 mmHg). BPs (129 ± 4/86 ± 3 mmHg) of the rats on standard rat chow during the first two weeks in each group were in the normal range. The high salt diet increased both SBP and DBP within three weeks and continued to raise the BPs to moderate hypertension by five weeks (150 ± 4/106 ± 3 mmHg vs. 129 ± 4/86 ± 3 mmHg, week 7 vs. week 2; *P* < 0.05, for both SBP and DBP; *n* = 36). The significant increases in BPs after a high salt diet for five weeks (e.g., at week 7) were similar among the three hypertensive groups (hypertensive control, sham-EA, EA). The elevated BPs in the untreated hypertensive (*n* = 8) and sham-EA-treated (*n* = 14) rats were similar throughout week 7 to week 11. In contrast, EA at ST36-37 lowered the high SBP and DBP (*n* = 14). EA's effect started one week after two treatment sessions and remained markedly low throughout the remaining 3-week treatment relative to the sham-EA treatment and untreated hypertensive controls (all *P *< 0.05; see Figure 2 for details). After three weeks of EA's therapy, BP was reduced close to prehypertension (prior to week 5) in the DSHRs (*P* > 0.05; *n* = 14). BPs of rats in the three hypertensive groups were higher than those in the normotensive control group from week 5 (after three weeks of the salt diet) to week 11 (the end of the experiment). Heart rate was unchanged in all groups over the 11 weeks.

### Manipulating A_2A_R activity in normotensive DS rats through blockade and activation

We performed rVLM microinjection of SCH 58261 or CGS-21680 unilaterally following BP observations for 11 weeks in normotensive control DS rats (without EA or sham-EA treatments). As demonstrated in Table 1, we noted that the administration of SCH 58261 (1 mM, 50 nl) or its vehicle (DMSO) into different sites of the rVLM in random order did not alter BP in four DS rats, which was noted as 125 ± 5/76 ± 7 and 125 ± 6/76 ± 6 mmHg (*P* > 0.05; *n* = 4), before and after SCH 58261, respectively. In four separate DS rats, unilateral microinjection of CGS-21680 into the rVLM decreased SBP and DBP very slightly. But there was no significant difference compared to the basal BP (123 ± 3/68 ± 3 vs. 120 ± 3/65 ± 2 mmHg, before vs. after CGS-21680; *P* > 0.05; *n* = 4) in the normotensive rats. The administration of the vehicle (DMSO) for CGS-21680 into other rVLM sites of these rats did not alter BP. Baseline BP is established prior to any microinjection. Heart rate was not affected by either A_2A_R blockade or stimulation in the DS normotensive rats.

**Table 1:** Blood pressure in response to blocking and stimulating A_2A_R in the rVLM of normotensive DS rats.

Injected drugs	Before the injection		After the injection	
	SBP (mmHg)	DBP (mmHg)	SBP (mmHg)	DBP (mmHg)
SCH 58261 (*n* = 4)	125 ± 5	76 ± 7	125 ± 6	76 ± 6
DMSO (*n* = 4)	120 ± 6	74 ± 3	119 ± 6	74 ± 4
CGS-21680 (*n* = 4)	123 ± 3	68 ± 3	120 ± 3	65 ± 2
DMSO (*n* = 4)	121 ± 2	64 ± 2	120 ± 2	64 ± 3

Means ± SE. SCH 58261 (1 mM, 50 nl) or its vehicle (DMSO) were administered unilaterally into different sites of the rVLM in random order in four Dahl salt-sensitive (DS) rats. In four separate DS rats, CGS-21680 or its vehicle (DMSO) were randomly microinjected unilaterally into different sites of the rVLM. There was no significant difference in SBP and DBP before and after the injection of each drug or its vehicle into the rVLM.

### A_2A_R and decrease in BP in DSHRs by EA

#### A_2A_R antagonism in EA-treated DSHRs

**Figure 3:**
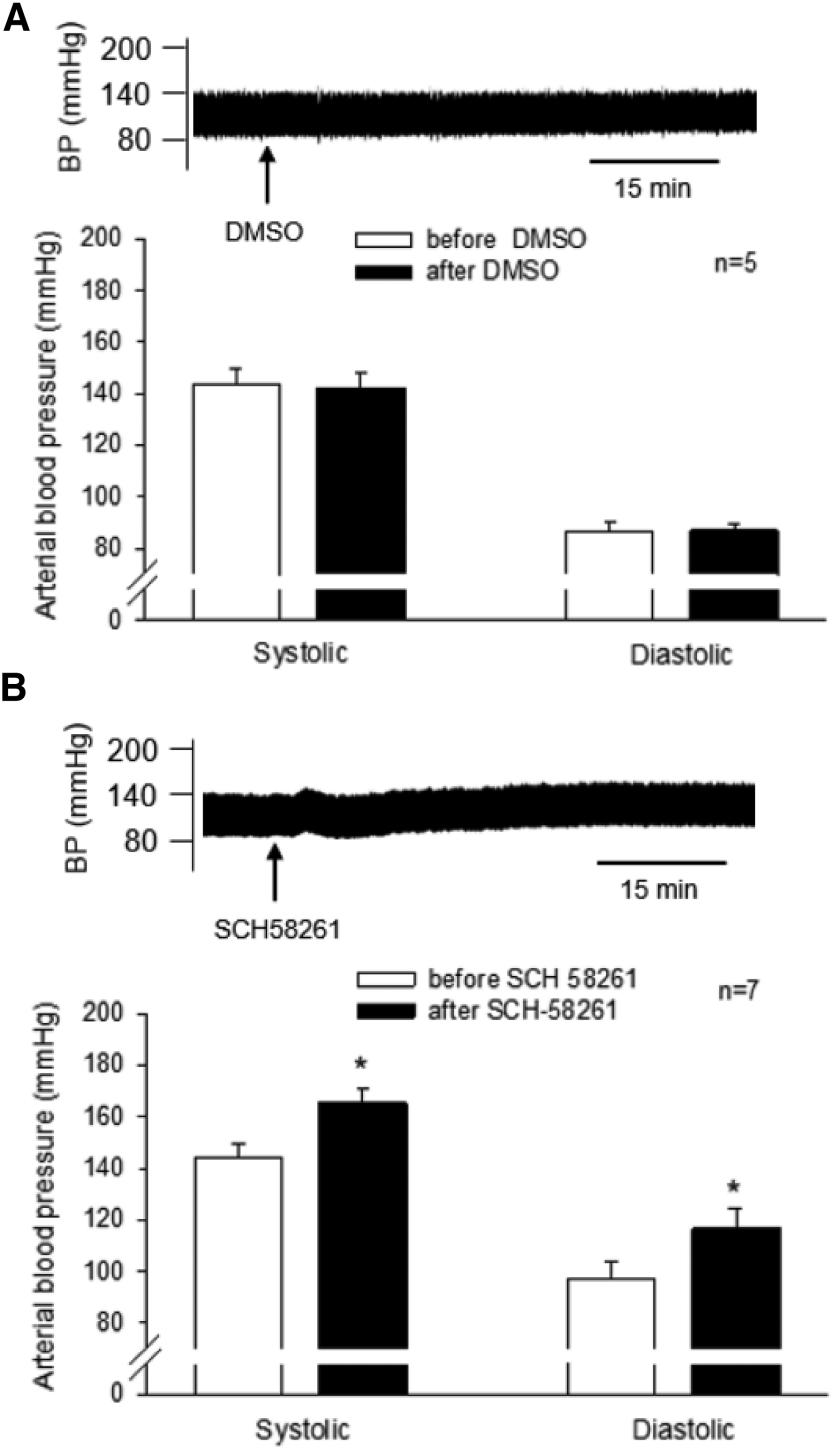
Involvement of A_2A_ receptors (A_2A_R) in the rVLM in EA modulation of high blood pressure (BP) in dahl salt-sensitive hypertensive rats (DSHRs). (**A**) and (**B**) changes in BP induced by the microinjection of DMSO (the vehicle for SCH 58261; Panel **A**) or SCH 58261 (an A_2A_R antagonist; 1 mM, 50 nl; Panel **B**) into the rVLM of EA-treated DSHRs. In (**A,B**), examples of original BP tracings before and after the microinjection are displayed above the bar graphs. Arrows below the tracing in (**A,B**) indicate the time of the microinjection into the rVLM.

We observed that the unilateral administration of SCH 58261 (1 mM, 50 nl) into the rVLM increased SBP and DBP, reversing EA's effect on BP (Figure 3). The peaks of SBP and DBP after SCH 58261 administration were significantly higher than their baseline levels (all *P* < 0.05, *n* = 7; Figure 3). In contrast, the vehicle (DMSO) for SCH 58261 microinjected into five of these seven animals did not change BP (Figure 3). Heart rate was not influenced by SCH 58261 or its vehicle in these DSHRs.

#### A_2A_R activation in DSHRs treated with sham-EA

**Figure 4:**
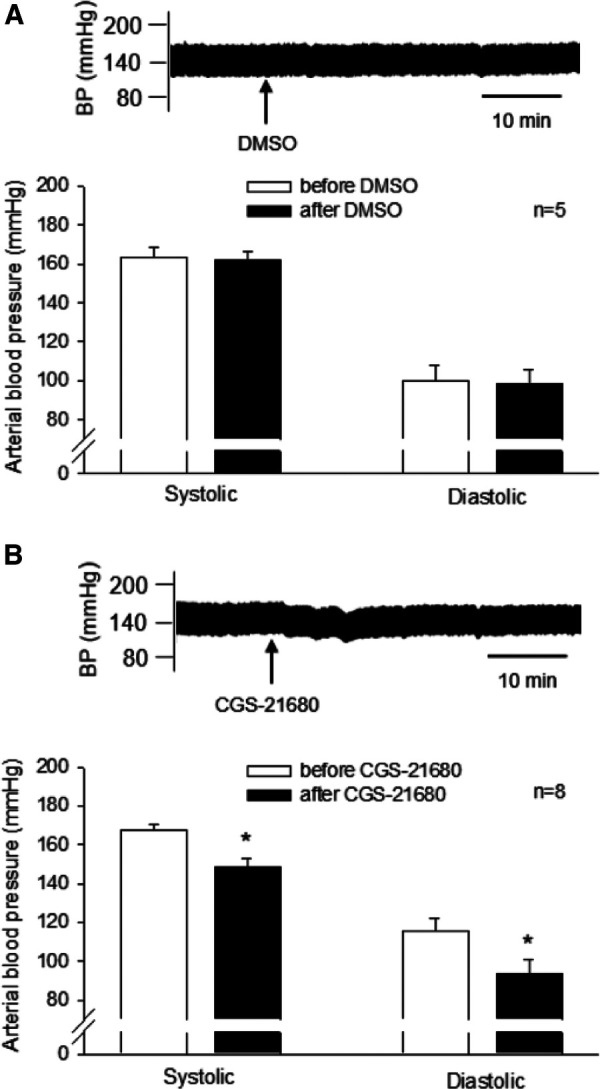
Influence of activating A_2A_ receptors (A_2A_R) in the rVLM on high blood pressure (BP) in dahl salt-sensitive hypertensive rats (DSHRs) treated with sham-EA. (**A,B**) Alterations in BP following the microinjection of DMSO (the vehicle for CGS-21680; Panel **A**) or CGS-21680 (an A_2A_R agonist; 0.4 mM, 50 nl; Panel **B**) into the rVLM of DSHRs treated with sham-EA. * *P* < 0.05, after vs. before the microinjection. In (**A,B**), examples of original BP tracings before and after the microinjection are displayed above bar graphs. Arrows below the tracing in (**A,B**) indicate the time of the microinjection into the rVLM.

CGS-21680 (0.4 mM, 50 nl) or vehicle was microinjected unilaterally into the rVLM of DSHRs treated with sham-EA. The vehicle for CGS-21680, DMSO (50 nl), was administered into unilateral rVLM in five of the eight rats. DMSO did not alter SBP and DBP (*n* = 5; Figure 4, Panel A). The agonist microinjection markedly decreased SBP and DBP (Figure 4, Panel B). The decreased BP lasted for over 30 min. The peak reductions in SBP and DBP following CGS-21680 microinjection were significantly lower than their baselines, prior to the activation of the A_2A_R (all *P* < 0.05, *n* = 8; Figure 4, Panel B). Heart rate was unchanged following the administration of either one of the chemicals.

### Microinjection sites

**Figure 5:**
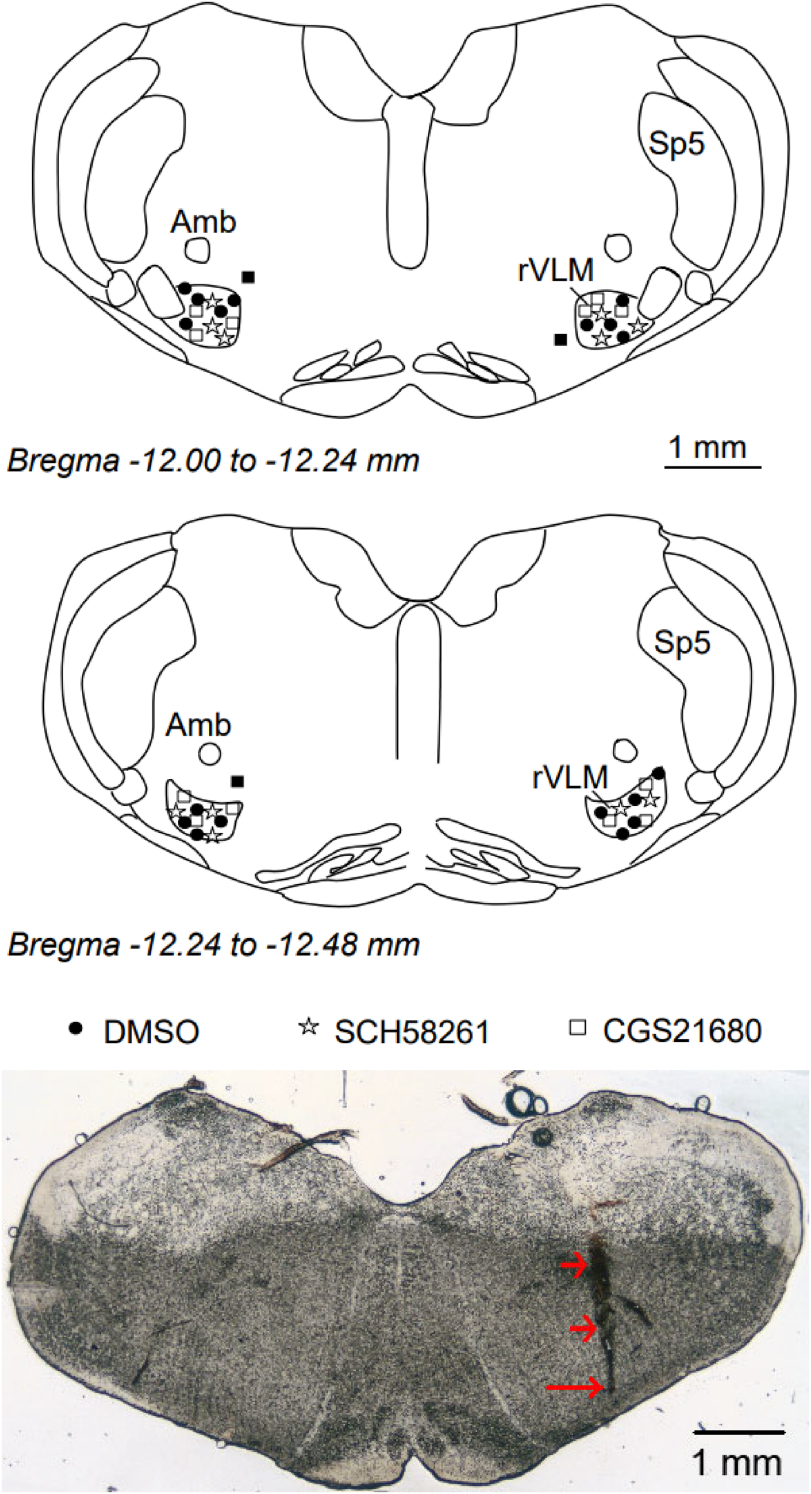
Anatomic locations of microinjection sites in the rat. Top and middle panels: Composite maps displaying histologically verified sites of microinjections in the rVLM of Dahl salt-sensitive rats. The brain section shows the composite of planes of the medulla oblongata (Paxinos and Watson's atlas). Symbols represent the microinjection of DMSO (the vehicle, ●), SCH 58261 (⋆), CGS-21680 (□), and injections outside the rVLM (▪). All microinjections were performed unilaterally, with the sides chosen at random. rVLM, rostral ventrolateral medulla; Sp5, spinal trigeminal nucleus; Amb, nucleus ambiguus. Bottom panel: An original slide of the medulla oblongata (Bregma −12.12 mm) shows a track of a microdialysis probe insertion used for injections, indicated by two short arrows. A long arrow indicates the site of the microinjection in the rVLM.

We examined the rat's brain slices after the microinjection of SCH 58261, an A_2A_R antagonist, or CGS-21680, an A_2A_R agonist, into the rVLM. We verified that each of the injections was located within the rVLM, except for three injections that were found to be outside the rVLM. These three injections included two injections of SCH 58261 and one injection of CGS-21680 in the hypertensive rats. None of the injections outside the rVLM influenced the BP. They served as the site control for rVLM microinjection and were not included in the statistical analysis and data presentation. Microinjection sites considered to be within the rVLM were observed to be confined by Bregma −12.48 to −12.00 mm (1.92–2.40 mm rostral to the obex), 0.5–1.0 mm from the ventral surface, and 1.8–2.3 mm lateral to the midline (Figure 5), as noted in the atlas of Paxinos and Watson (33).

### Increase in rVLM A_2A_R expression by EA in DSHRs

**Figure 6:**
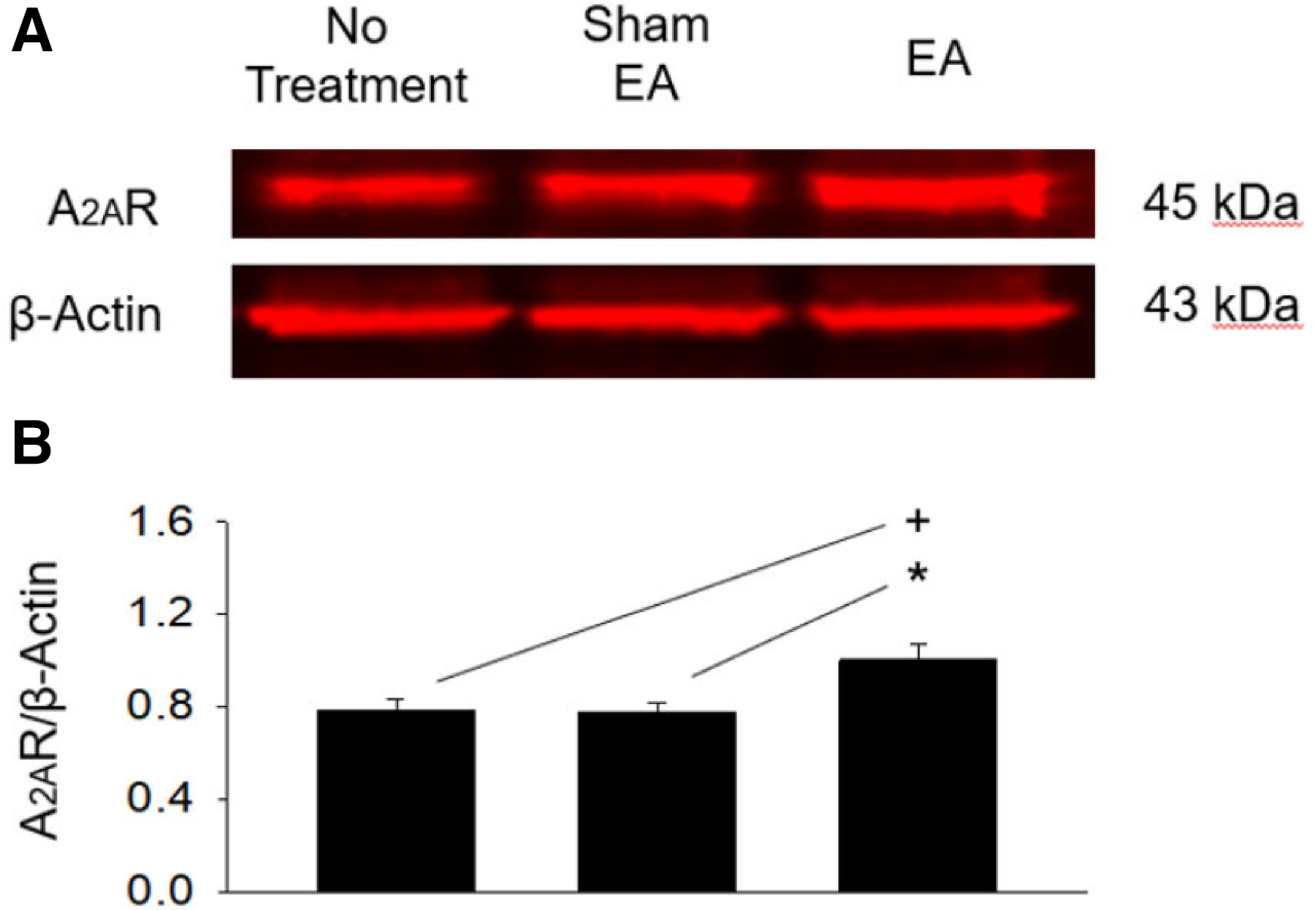
Protein expression of A_2A_ receptors in the rVLM of dahl salt-sensitive hypertensive rats. (**A**) Original bands display protein levels of A_2A_ receptors (A_2A_R) in the rVLM from three rats, one from each hypertensive experimental group. (**B**) Group data show that the protein expression of rVLM A_2A_R 24 h after terminating EA was increased relative to controls and sham-EA. The relative value of A_2A_R/*β*-Actin is shown in the bar graph (*n* = 5 for each group). Symbols +, * indicate significant differences (*P* < 0.05) compared respectively with hypertensive controls and animals treated with sham-EA.

A_2A_R protein expression in the rVLM of the EA group (*n* = 5) was increased significantly (all *P* < 0.05; Figure 6) following EA treatments compared to that in the groups subjected to untreated hypertensive control (*n* = 5) and sham-EA (*n* = 5). There was no significant difference in rVLM A_2A_R expression among sham-EA and untreated hypertensives.

## Discussion

The present study demonstrates several significant findings. First, unilateral activation or inhibition of rVLM A_2A_R in normotensive control DS rats does not alter BP and enables the study of A_2A_R involvement in the actions of EA in hypertensive rats. Second, real EA but not sham-EA treatment at ST36-37 decreases BP in DSHRs. Third, the decreased BP after eight treatments by EA at ST36-37 in DSHRs is reversed by unilateral blocking the A_2A_R in the rVLM with antagonist SCH 58261. Fourth, in DSHRs treated with sham-EA, unilateral rVLM administration of an A_2A_R agonist, CGS-21680, reduces BP. Fifth, the A_2A_R expression is increased in the EA-treated DSHRs but not with sham-EA treatment when compared to hypertensive controls. Our results imply that an 8-session application of EA at ST36-37, a non-pharmacological treatment, can reduce high BP in hypertensive rats, likely through adenosine-A_2A_R mechanisms in the rVLM.

We and others have demonstrated that acupuncture can decrease BP in experimentally hypertensive models such as the spontaneously hypertensive rat, stress- and cold-induced hypertensive rats (4, 31, 38–40). In about 20%–30% of these studies, the ST36 acupoint was applied (38, 39). DS rats fed with a high-salt diet, shown in our recent study, are another model used commonly to study hypertension since DS rats with hypertension mimic many characteristics of essential hypertension in patients, including increased sympathetic activity (17, 18). Increased rVLM pre-sympathetic neuronal and sympathetic nerve activities are inhibited by EA (5, 20, 41, 42). In the present study, we found that EA (ST36-37) but not sham-EA reduced high BP in DSHRs, similar to that observed in other hypertensive models and patients with essential hypertension (4, 19, 39). Our findings show that the EA-induced BP-lowering effect in DSHRs is similar to other hypertensive models, but the central EA action is yet unclear. This further supports EA's beneficial effect on hypertension and begs the quest for the in-depth understanding of EA's neuromodulatory mechanisms.

In a previous publication (42), we demonstrated convergent input in rVLM neurons resulting from somatic afferent activation during EA stimulation through rVLM recordings experiments. Moreover, EA applied at ST36-37 inhibits the activity of rVLM premotor neurons and sympathoexcitatory cardiovascular responses (34, 42, 43). These effects of EA at ST36-37 are similar to those of EA stimulation at the P5-6. Congruent with the findings of Sato et al. showing that Group I afferent fibers do not affect cardiovascular functions (44), our research has extensively demonstrated that the use of low-frequency and low-intensity EA at P5-6 activates finely myelinated (Group III) and unmyelinated (Group IV, C-fibers) afferents through directly depolarizing them via electric current applied in the region of the acupoints, subsequently eliciting somatic-sympathetic modulatory responses (32, 43, 45, 46). These findings support that EA at ST36-37 likely stimulated Group III and IV afferent fibers, which send inputs to the brain and modulate rVLM activity and cardiovascular function (47).

Adenosine is formed from ATP following neuronal activation and is considered a neuromodulator in the nervous system (11, 12). Of the four subclassified adenosine receptors (A_1_, A_2A_, A_2B_, and A_3_) present in the body, the A_2A_R is a predominant subtype of adenosine receptor expressed in the rVLM, which participates in modulating cardiovascular responses (14, 48). A previous study has demonstrated that the application of adenosine into the rVLM caused a short-lasting increase or a long-lasting depression of rVLM neuronal activity (48). We and others have observed that microinjection of a non-selective adenosine receptor blocker (e.g., 8-sulphophenly-theophline) into the rVLM did not affect the baseline levels of BP in normotensive rats, but attenuated excitatory cardiovascular responses with increased BP, suggesting that adenosine-mediated receptors are unlikely to play an essential role in controlling basal BP but modulate high BP associated with increased sympathetic activity observed in sympathoexcitatory reflex responses (13, 14).

The present study shows that unilateral activation of specific A_2A_R (microinjection of agonist CGS-21680) does not influence BP in our normotensive rats. However, the activation of this receptor unilaterally decreased visceral sympathoexcitatory BP reflex responses. These results imply that A_2A_R in the rVLM is likely associated with modulating elevated sympathetic nerve activity and increased BP (13). Moreover, we have found that unilaterally antagonizing rVLM A_2A_R does not alter the basal BP but reverses EA's modulatory effect on sympathoexcitatory pressor responses (13). These findings suggest the involvement of rVLM A_2A_R during EA-inhibition of sympathoexcitatory-induced transient elevated BP in normal rats. Notably, EA does not alter BP in normotensive conditions (26–28). Thus, these previous observations provide a rationale to examine the A_2A_R mechanism contributing to the BP-lowering effect of EA (ST36-37) in DSHRs, which have not been investigated. In the present study in rats with chronic elevation of BP, we show that unilateral microinjection of the A_2A_R antagonist into the rVLM reverses the BP-lowering effect of EA treatment in salt-sensitive hypertensive rats. The unilateral administration of this antagonist does not influence BP in normotensive rats, implying further that A_2A_R may not chiefly contribute to the regulation of basal BP. In addition, present findings show that mimicking the effect of EA (ST36-37) with the A_2A_R agonist decreases the high BP in sham-EA hypertensive rats. Our data indicate that compared to normotensive conditions, A_2A_R in the rVLM in hypertension likely overall is increased in activation in response to its exogenous agonist and endogenous ligand, adenosine, generated following acupuncture stimulation (49). Altogether, activating rVLM A_2A_R is likely important in rebalancing the elevated BP, in both acute and chronic hypertensive conditions, especially following EA treatment.

Interestingly, present novel data indicates significantly increased A_2A_R expression in the rVLM following EA treatment. We have found that repetitive application of EA but not sham EA twice a week over four weeks lowered elevated BP as well as increased A_2A_R expression in the rVLM of hypertensive DS rats at the end of the treatments relative to hypertension controls. Previous studies have shown that increased A_2A_R expression parallels an enhanced level of adenosine in the rVLM. The function of high A_2A_R expression in the rVLM is associated with hypotensive responses (36, 37). As such, the data from the present study suggests that the stimulation of deep peroneal nerve with EA at ST36-37 lowers high BP in DSHRs concomitantly with an increased A_2A_R expression in the rVLM. The above observations imply that EA-associated increase in A_2A_R expression (and possibly increased adenosine) enhances the action of rVLM adenosine-A_2A_R mechanism to, in turn, decrease sympathetic outflow and, ultimately, hypertension. Thus, the increased expression of A_2A_R findings provides new insight into the mechanisms underlying EA's effects on hypertension. A possible mechanism to increase A_2A_R by EA may be related to the fact that EA likely causes neuronal activation and generates adenosine in the rVLM, as acupuncture induces the production of adenosine at ST36 (49), which subsequently leads to the upregulation of adenosine A_2A_R and modulates the activity of cardiovascular-related neurons (37, 50). Other complex mechanisms may also participate in EA-induced elevation in A_2A_R expression. In this respect, one previous study showed that adenosine and A_2A_R levels increased in the rVLM following L-arginine microinjection, suggesting that nitric oxide may increase A_2A_R in the rVLM (37). Another study reported that the BP-lowering effects of EA at ST36 on stress-induced hypertension are related to increased inducible nitric oxide synthase in the rVLM of rats (51). As such, EA likely can enhance A_2A_R expression through nitric oxide as well, in addition to upregulating this kind of receptor via its endogenous ligand, adenosine.

Studies performed on animals have allowed for a deeper understanding of the mechanisms by which EA exerts its modulatory effects on elevated BP. The findings from the present study suggest that the A_2A_R participates in the central processing of EA's action in lowering BP in hypertension at the end of treatment. Additional studies are warranted if A_2A_R plays a role during the course of treatment, i.e., onset of the EA-induced BP lowering effect and continual decrease of BP. Moreover, we may further examine if the A_2A_R interacts alongside other neural substances in the rVLM, such as GABA, to reduce BP following EA treatment. It is also worthwhile to examine if A_2A_R in other brain regions, such as the hypothalamic paraventricular nucleus that is involved in EA-induced BP reduction (52). Besides, our previous clinical study showed that EA BP-lowering effects in patients with hypertension remained for a few weeks after terminating EA treatment, suggesting EA's prolonged effect on hypertension (19). It is uncertain if A_2A_R in the central nervous system contributes to the long-lasting action of EA in lowering BP in hypertensive conditions. Future studies are warranted to understand the mechanisms underlying EA's hypotensive effects further.

### Perspective

Hypertension remains a significant cause of morbidity and mortality in individuals, particularly aged patients with cardiovascular diseases. Since ideal control of hypertension is challenging using regular medications due to the incompliance, it is critical to search for alternative or integrative ways to manage hypertension well. Acupuncture has potential advantages over conventional medical therapy, including prolonged action and rare side effects (5, 19). However, its clinical application is limited due to the need for robust scientific evidence supporting its effectiveness and the underlying mechanisms (53). Thus, there is a critical need for rigorous research to improve our understanding of acupuncture's effect on hypertension. The present study uncovered adenosine's role through rVLM A_2A_R in EA modulation of elevated BP in salt-sensitive hypertension, like essential human hypertension. The new findings expand our understanding of neural mechanisms underlying EA's BP-lowering effect on hypertension. As such, our previous and current studies suggest that acupuncture can be used as an alternative and integrative regimen for managing hypertension.

The efficacy of EA in lowering BP may be significant for patients with advancing age (>60 years) since spikes in BP place aged patients with hypertension at great risk for stroke, myocardial infarction, and other cardiovascular diseases. Thus, reducing BP with EA has the potential to decrease the risk for these cardiovascular disorders.

In summary, the data from the present study demonstrated that the A_2A_R in the rVLM may contribute to the BP-lowering effect of 8-session EA therapy in hypertension. The results provide further insight into the mechanisms underlying EA's ability to decrease elevated BP. Moreover, our new findings allow us to expand our knowledge of non-pharmacological regimens for hypertension management to provide more additional healthcare.

## Data Availability

The raw data supporting the conclusions of this article will be made available by the authors, without undue reservation.
